# Fatty Acid Signaling Impacts Prostate Cancer Lineage Plasticity in an Autocrine and Paracrine Manner

**DOI:** 10.3390/cancers14143449

**Published:** 2022-07-15

**Authors:** Le Zhang, Sandrine Billet, Gabrielle Gonzales, Krizia Rohena-Rivera, Hayato Muranaka, Gina Chia-Yi Chu, Qian Yang, Hyung Kim, Neil A. Bhowmick, Bethany Smith

**Affiliations:** 1 Department of Medicine, Cedars-Sinai Medical Center, Los Angeles, CA 90048, USA; le.zhang@cshs.org (L.Z.); sandrine.billet@cshs.org (S.B.); gabrielle.gonzales@cshs.org (G.G.); krizia.rohena-rivera@cshs.org (K.R.-R.); hayato.muranaka@cshs.org (H.M.); chucy88@gmail.com (G.C.-Y.C.); qian.yang@cshs.org (Q.Y.); hyung.kiml@cshs.org (H.K.); 2 Department of Research, VA Greater Los Angeles Healthcare System, Los Angeles, CA 90073, USA

**Keywords:** cholesterol, free fatty acid, Wnt, hedgehog, cancer associated fibroblast, prostate cancer, androgen

## Abstract

**Simple Summary:**

A high-fat diet is implicated in prostate cancer progression in patients. Prostate-cancer-associated fibroblasts play an important role in promoting tumor progression and therapeutic resistance to androgen-receptor-signaling inhibitors, such as enzalutamide. We investigated the mechanism of saturated fatty acids’ impact on prostate cancer reprogramming. Our work demonstrates that the tumor microenvironment defines the biology of prostate cancer progression induced by saturated fatty acids. This study also provides relevant data to potentially improve prognosis for patients with high fat intake through the inhibition of the identified signaling pathways.

**Abstract:**

Prostate cancer (PCa) affects an estimated 250,000 men every year and causes 34,000 deaths annually. A high-fat diet and obesity are associated with PCa progression and mortality. This study’s premise was the novel observation of crosstalk between PCa epithelia and cancer-associated fibroblasts (CAF) in response to palmitate-mediated lineage plasticity. We found that cholesterol activated canonical Hedgehog (Hh) signaling by increasing cilium Gli activity in PCa cells, while palmitate activated Hh independent of Gli. Exogenous palmitate activated SOX2, a known mediator of lineage plasticity, in PCa cells cocultured with CAF. Stroma-derived Wnt5a was upregulated in CAF while cocultured with PCa cells and treated with palmitate. Wnt5a knockdown in CAF inhibited Hh and SOX2 expression in PCa cells from cocultures. These findings supported our proposed mechanism of a high-fat diet promoting Hh signaling-mediated transformation within the tumor microenvironment. SOX2 and Wnt5a expression were limited by the CD36 neutralizing antibody. Mice xenografted with PCa epithelia and CAF tumors were fed a high-fat diet, leading to elevated SOX2 expression and lineage plasticity reprogramming compared to mice fed an isocaloric rodent diet. CD36 inhibition with enzalutamide elevated apoptosis by TUNEL, but limited proliferation and SOX2 expression compared to enzalutamide alone. This study revealed a mechanism for a high-fat diet to affect prostate cancer progression. We found that saturated fat induced lineage plasticity reprogramming of PCa by interaction with CAF through Wnt5a and Hh signaling.

## 1. Introduction

Prostate cancer is expected to reach approximately 2 million new cases by 2040, with the United States (US) having the highest incidence rate of prostate cancer (PCa) worldwide [1]. A high-fat diet and obesity are identified as important drivers of disease progression and accelerated patient mortality [2,3,4,5,6]. Since a third of the US population is categorized as obese [7], the underlying mechanisms of this phenomenon require immediate attention. The ingestion of a high-fat diet is associated with the elevated circulation of free fatty acids [8]. Palmitic acid (palmitate) is a common saturated long-chain fatty acid and a significant component of the Western-style diet that is metabolized to cholesterol by the prostate and other organs, including the liver [9]. Liver and breast cancers are among the many diseases particularly associated with the elevated oxidative stress resulting from palmitate consumption [10]. We investigated the impact of palmitate and cholesterol supplementation on the interaction between PCa epithelia and prostate-cancer-associated fibroblasts (CAF).

Cholesterol signaling has been associated with cancer development, organogenesis, and progression through the Hedgehog (Hh) signaling pathway [11]. The contribution of Hh signaling to the regulation of cancer stemness and invasiveness has been demonstrated in many cancer types, including prostatic, pancreatic, ovarian, and colorectal [12,13,14,15]. The connection of Hh signaling and SOX2 has implications on PCa cell viability and androgen-independence [16]. Past reports of the regulation of castration-resistant PCa (CRPC) demonstrated Hh signaling inhibition sensitized CRPC to androgen-targeted therapy [17,18,19,20]. Interestingly, Hh signaling target genes are expressed nearly exclusively by colon fibroblast cells of the colon cancer microenvironment, while cognate ligands were expressed by tumor cells [21]. Reports have indicated that lipids can regulate Hh signaling at multiple levels, and that cholesterol modifications needed to support the interactions of Hh with the cell membrane promote Hh protein stability in the extracellular milieu [11,22,23]. However, the involvement of Hh signaling in the interactions between PCa epithelium and stroma in the context of a high-fat diet remains an open question.

Stromal–epithelial interactions play a role in cancer progression, differentiation, and therapeutic responsiveness [24]. CAF are activated fibroblasts that have an elevated secretion of chemokines, matrix proteins, and immunomodulatory factors compared to normal fibroblasts [25,26,27,28,29]. The volume of CAF in PCa validated by the tissue microarray of 847 patients is a suggested prognostic marker for recurrence-free survival [30]. CAF and normal fibroblasts can be functionally differentiated as benign cells that can induce tumorigenesis in non-tumorigenic prostatic epithelia, as previously reported [31,32,33]. The interaction of cancer and stromal fibroblast cells contribute to PCa progression at both early and late stages of the disease [34,35]. We have previously published that stromal epigenetic alterations mediate paracrine oncogenic signaling and epithelial metabolic reprogramming, along with altering sensitivity to androgen-targeted therapy [36,37,38,39,40,41,42]. The implications of lipids in paracrine signaling was demonstrated recently as CAF were found to secrete fatty acids and phospholipids in potentiating colorectal cancer-cell migration [43]. Further, the intake of saturated fatty acids has been associated with PCa progression [44]. As with other cancers, the lineage plasticity of PCa is defined as a morphologic and functional shift from adenocarcinoma to another differentiation state in response to therapy, often having features of a small cell or neuroendocrine phenotype [45]. Here, we examine a mechanism that explains the observed cooperative signaling of saturated fatty acids and cholesterol in promoting PCa lineage plasticity. SOX2 has emerged as an important mediator of PCa lineage plasticity, with this differentiative state being associated with androgen-targeted-therapy resistance [46]. Blocking saturated fatty acid signaling in CAF limited paracrine-mediated PCa progression in combination with enzalutamide, a widely used second-generation androgen-receptor-signaling inhibitor (ARSI).

## 2. Materials and Methods

### 2.1. Reagents

Enzalutamide (Pfizer, New York, NY, USA) was given to cells at 5 uM and 5 mg/kg in mice by oral gavage. CD36 neutralizing antibody, clone: FA6-152 (NB6001423, Fisher Scientific, Waltham, MA, USA) was administered at 0.1 mg/kg to mice by intraperitoneal injection, and at 2 µg/mL in cultured cells. Mouse IgG1, clone: NCG01, isotype control (PIMA514453, Fisher Scientific) was given to mice at 0.1 mg/kg by intraperitoneal injection. Palmitic acid (P0500, Sigma, Darmstadt, Germany) was given to cells at 50 µM, 100 µM, 150 µM, and 200 µM. Cholesterol (C75209, Sigma, St Louis, MO, USA) was given to cells at 20 µg/mL. GANT61 (G9048, Sigma) was given to cells at 5 µM. Simvastatin (AC458840010, Fisher Scientific) was administered to cultured cell at 5 nM. Cells were treated for 48 h for each drug.

### 2.2. Cell Lines

Human prostate tumor cell line CW22Rv1 (22Rv1) was purchased from ATCC and grown in RPMI-1640 supplemented with either 10% fetal bovine serum (FBS) or 3% charcoal-stripped FBS (for palmitic acid and cholesterol treatment where indicated), and 1% penicillin/streptomycin (all components from Thermo Fisher). Human androgen-refractory prostate cancer cells of the ARCaP_M_ cell line were gifted to us from Leland Chung (Cedars-Sinai Medical Center), and grown in DMEM, supplemented with 5% FBS and 1% penicillin/streptomycin [24].

Human primary fibroblasts were grown from prostatectomy specimens at Cedars-Sinai Medical Center or the Greater Los Angeles Veterans Affairs under their respective institutional review boards [42]. The tumor-inductive status of fibroblasts was determined by tissue recombination with BPH1 (a non-tumorigenic human prostate epithelial cell line, as previously described [32]). CAF was cultured in DMEM/F12 supplemented with 5% FBS, 5% Nu-Serum, 1% penicillin/streptomycin, 10^−9^ M testosterone (Sigma-Aldrich), and 4 μg/mL insulin (12585014, Fisher Scientific). All cells were grown in a humidified incubator at 37 °C with 5% CO_2_. All cells were tested for mycoplasma (LT07118, Lonza, Rockland, ME, USA) every 1 month and were negative.

### 2.3. RNA Preparation, cDNA Synthesis, qPCR

Total RNA was extracted with RNeasy Plus Mini kit (74034, Qiagen, Hilden, Germany) according to the manufacturer’s recommendations. RNA levels were measured with a NanoDrop spectrophotometer (Thermo Fisher) at 260 nm. cDNA synthesis was performed on 1 μg of total RNA using iScript cDNA Synthesis Kit (1708891, Bio-Rad, Hercules, CA, USA). Quantitative real-time PCR reactions were performed using SYBR Green Mix (Azura Genomics, Raynham, MA, USA). qPCR reactions were performed on 96 well qPCR plates using Thermos qPCR systems (Thermo Fisher Quant Studio3) according to manufacturer’s instructions. Data were calculated as relative mRNA expression to indicated housekeeping gene (2^−ΔΔCt^). Results were obtained from at least three independent experiments and are shown as the mean ± SD. Primers were purchased from IDT (Coralville, IA, USA). Please refer to Supplementary Table S1 for the primers used with sequences.

### 2.4. Immunofluorescence

A total of 5 × 10^4^ cells were seeded on cover slips overnight, then treated with 20 µg/mL of cholesterol for 48 h. All subsequent steps were performed at room temperature. Cover slips were then fixed with 4% formaldehyde for 15 min. Following this, cells were permeabilized with 0.5% Triton-X100/PBS for 5 min. For immunostaining, cover slips were blocked with 10% serum/PBS for 1 h and washed with PBS. The cells were incubated with a combination of primary antibody α-acetylated α-tubulin (T7451, Sigma) and ß-catenin (sc-7199, Santa Cruz, Santa Cruz, CA) overnight at 4 °C. After washing twice with PBS for 5 min, the cover slips were incubated with fluorochrome-coupled secondary antibody (Alexa Fluor 488, A11034; Alexa Fluor 546, A11030; Fisher Scientific) for 2 h in the dark. After washing with PBS and rinsing with H_2_O, the cells were covered with a mounting medium containing DAPI (H-1200, Vector Laboratories, Burlingame, CA, USA). Stained cells were imaged on a Leica confocal microscope at 40X magnification (Leica Microsystems, Wetzlar, Germany).

### 2.5. Flow Cytometry

A quantity of 2 × 10^5^ cells were seeded in 6-well format overnight, then treated with 20 µg/mL of cholesterol for 48 h. Cells were washed with PBS, detached using Accutase solution (00455556, Fisher Scientific), fixed with 4% paraformaldehyde (IC fixation buffer, eBioscience, San Diego, CA, USA) at room temperature for 15 min on ice. Cells were permeabilized on ice with 1X permeabilization buffer (eBioscience) for 10 min. Cells were incubated with primary antibody α-acetylated α-tubulin (T7451, Sigma) for 30 min on ice. Cells were then washed twice with 1X PBS before 30 min of incubation with Alexa Fluor 488 secondary antibody (A11001, Fisher Scientific) on ice in the dark. Cells were washed twice with PBS, then resuspended in PBS and analyzed using a BD Accuri C6 flow cytometer (BD Biosciences, Franklin Lakes, NJ, USA). Cells were kept on ice and in the dark until ready for analysis. FACS data were analyzed using FlowJo software v10.9.

### 2.6. Chromatin Immunoprecipitation (ChIP) Sequensing

A quantity of 1 × 10^7^ cells were harvested and fixed with 2 mL of 1% paraformaldehyde for 10 min at room temperature with gentle rotation. A total of 2 mL of 250 mM of glycine was then added to terminate the processing. A ChIP experiment for H3K27Ac (ab4729, Abcam, Cambridge, UK) and a computational analysis of ChIP-seq data were performed as previously described [47].

### 2.7. Small-Interfering RNA (siRNA) Transfection

Cells at 70% confluence were transfected with 25 nM Wnt5a siRNA (sc-41112, Santa Cruz) and control siRNA (sc-37007, Santa Cruz) using Lipofectamine 3000 (Fisher Scientific), as described by the manufacturer. Twenty-four hours after the addition of the transfection mix, the liposomes were removed and fresh media was added. The human Wnt5a siRNA is a pool of 3 different siRNA duplexes:

A: Sense: GCAAGUUGGUACAGGUCAATT,
antisense: UUGACCUGUACCAACUUGCTT;
B: Sense: GACAGACCGUCAUAUUCUATT,
antisense: UAGAAUAUGACGGUCUGUCTT;
C: Sense: CCAGUGUACUUGAACAGUUTT,
antisense: AACUGUUCAAGUACACUGGTT.

### 2.8. Clonogenic Assays

Cells were seeded in 12-well plates overnight (22Rv1 700 cells/well, ARCaP_M_ 200 cells/well), then treated with palmitate, cholesterol, or both. Colonies were stained with 5% crystal violet in methanol for 20 min after 14 days. We then used 30% acetic acid in water solubilizing the colonies. The quantification of the colonies was shown by optical density 595 (OD595) measured by spectrophotometer in three independent experiments.

### 2.9. Animal Studies

All animal procedures were performed according to an approved protocol from the Institutional Animal Care and Use Committee at Cedars-Sinai Medical Center. Male NSG mice (Jackson Labs, Bar Harbor, ME, USA), 6–8 weeks old, were used for prostatic orthotopic grafting or subcutaneous implantations into the flank, as previously described [41]. For the orthotopic grafting, mice were randomly divided into 3 groups, fed a normal isocaloric rodent diet, a 40% high-fat diet (F10046, Bio-Serv, Flemington, NJ, USA), and a 2% high-cholesterol diet (F10036, Bio-Serv), respectively. The formulation of the high-fat and high-cholesterol diet are in Supplementary Table S2. After 4 weeks of each diet, 2.5 × 10^5^ ARCaP_M_ cells and 7.5 × 10^5^ CAF were engrafted orthotopically into the anterior lobe of the prostate and harvested after 4 weeks of implantation, as has been described before [36]. For the subcutaneous implantations, all mice were fed with a 40% high-fat diet (F10046, Bio-Serv) for 4 weeks, then 2.5 × 10^5^ 22Rv1 cells and 7.5 × 10^5^ CAF were implanted subcutaneously into the flank of the mice. After 4 weeks, mice were randomized to either be injected intraperitoneally with 0.1 mg/kg of neutralizing anti-CD36 antibody or corresponding IgG1 control every 3 days for a week. All mice were subsequently treated daily with enzalutamide for 3 days via oral gavage (5 mg/kg). Following these procedures, mice were euthanized and tumors were harvested for analyses.

### 2.10. Immunohistochemistry

All tissue biopsies were fixed in 4% paraformadehyde, embedded in paraffin, and cut into 5 μm sections. The tissue sections were deparaffinized and hydrated through xylene and a graded alcohol series using a standard protocol [42]. Following antigen retrieval, endogenous peroxidase activity was then quenched with 3% H_2_O_2_. Non-specific epitopes were blocked with blocking buffer for 1 h at room temperature. After washing with PBS, the sections were stained with anti-SOX2 (14962, Cell Signaling, Danvers, MA, USA) and anti-phosphorylated histone H3 (PH-H3, 06-570, Sigma) antibodies [48]. Sections were incubated at 4 °C overnight with adequate humidity. Sections were washed with PBS, then incubated with the appropriate secondary antibody (31460, Fisher Scientific) for 1 h at room temperature in a humidified chamber. Tissues were visualized by DAB (3,3′-diaminobenzidinetetrahydrochloride substrate, 550880, BD Biosciences). TUNEL staining was performed according to the manufacturer’s protocol (S7100, Fisher Scientific). Slides were scanned by Olympus BX51 at 200 x magnification. At least 5 fields per tissue were quantified with Image J.

### 2.11. Statistical Analysis

Comparisons between the studied groups were performed by the paired Student’s t-test. Two-way ANOVA was used to compare variable changes according to the levels of two categorical variables. Results were expressed as mean ± SD and considered statistically significant at *p* < 0.05. All data were calculated from at least 3 separate experiments. Graphs were prepared using GraphPad Prism 6 software (GraphPad Software, San Diego, CA, USA).

## 3. Results

### 3.1. Cholesterol and Fatty Acid Signaling in PCa Cells

**Figure 1:**
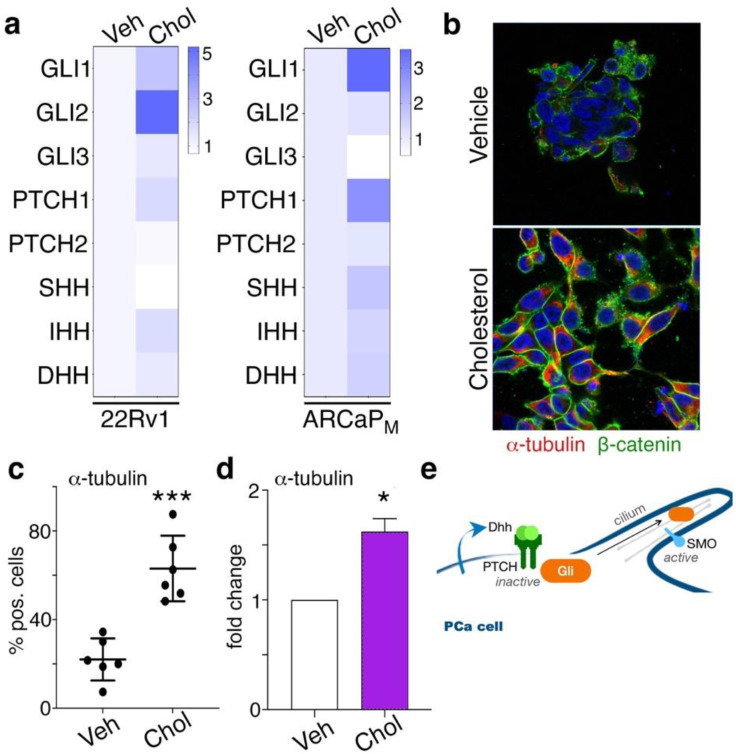
Cholesterol induces canonical Hedgehog signaling in PCa cells. (**a**) mRNA expression of Hh signaling mediators was measured in 22Rv1 and ARCaP_M_ treated with cholesterol. (**b**) 22Rv1 treated with cholesterol for 48 h was imaged by confocal microscopy (40× magnification). ß-catenin and α-tubulin were visualized by green and red fluorescence, respectively, with DAPI nuclear counter-stain (blue). (**c**) Image analysis for α-tubulin expression was performed by determining the percentage of positively stained cells in >30 field of view (FOV). (**d**) α-tubulin expression was detected by FACS in 22Rv1 treated with cholesterol. (**e**) Schematic of Hedgehog signaling at the primary cilium. DHH binding to PTCH, the inhibitor of Smo along the center of the cilium. For all figure panels, 22Rv1 and ARCaP_M_ cells were treated with 20 µg/mL cholesterol, or vehicle for 48 h. Paired, 2-tailed *t* test: * *p* < 0.05, *** *p* < 0.001.

Lowering cholesterol has been shown to limit PCa progression by providing a clinical benefit to patients [49,50]. Hh signaling in particular has been associated with cholesterol in PCa cells [11]. We corroborated past findings of Hh downstream gene expression in PCa cells following 48 h incubation with cholesterol (20 µg/mL). Cholesterol elevated Gli1 and Gli2 mRNA expression in 22Rv1 PCa cells (*p* < 0.05; Figure 1a). Cholesterol also increased Gli1 and PTCH1 mRNA expression in ARCaP_M_ PCa cells (*p* < 0.01; Figure 1a). The Hh signaling pathway depends on the secretion of the Hh ligand, Sonic Hh (SHH), Indian Hh (IHH), and Desert Hh (DHH). We detected these Hh ligands, and found that IHH was elevated in 22Rv1 (*p* < 0.01); IHH, SHH, and DHH were also elevated in ARCaP_M_ (*p* < 0.01). Two-way ANOVA analysis of these Hh-related genes in 22Rv1 and ARCaP_M_ treated with cholesterol were significantly increased compared to control cells (*p* < 10^−4^; Figure 1a). Canonical Hh signaling relies on primary cilia and is associated with α-tubulin expression [51]. We demonstrated for the first time in immunofluorescence studies that cholesterol-treated cells had greater cell-surface staining for α-tubulin by confocal microscopy and flow cytometry in 22Rv1 cells (*p* < 0.001; Figure 1b,c). This was quantified by quantitative PCR, where cholesterol treatment increased α-tubulin mRNA expression (*p* < 0.05; Figure 1d). Canonical Hh signaling involving patched expression-promoted Gli signaling was enriched at the cilium (Figure 1e).

**Figure 2:**
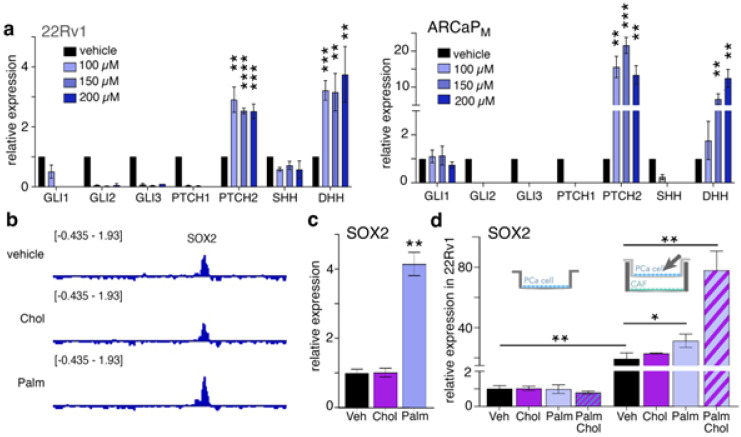
Palmitate induces Gli−independent Hedgehog signaling in prostate cancer cells. (**a**) Hh signaling gene mRNA expression in 22Rv1 and ARCaP_M_ treated with indicated palmitate concentrations. (**b**) Genome browser representations of H3K27ac ChIP−seq in 22Rv1 at the SOX2 loci. (**c**) SOX2 mRNA expression in 22Rv1 treated with cholesterol or palmitate. (**d**) 22Rv1 was either cultured alone or in a transwell with CAF as indicated. SOX2 mRNA expression in 22Rv1 treated with cholesterol, palmitate, or both in combination. For all figure panels, Hh signaling ligands or SOX2 mRNA expression in 22Rv1 and ARCaP _M_ cells were determined following treatment with 20 µg/mL cholesterol, 50 µM palmitate, a combination of 100 µM palmitate and 20 µg/mL cholesterol, or vehicle for 48 h. Results are normalized to 22Rv1 without CAF vehicle control (Veh). Paired, 2-tailed *t* test: * *p* < 0.05, ** *p* < 0.01, *** *p* < 0.001, **** *p* < 0.0001.

Following this, we wanted to determine the role saturated fatty acids play on Hh signaling in PCa cells independent of cholesterol signaling. We first evaluated the expression of Hh signaling in 22Rv1 and ARCaP_M_ cells. Both cell lines showed significantly more PTCH2 and DHH in response to palmitate treatment after 48 h and the absence of Gli and PTCH1 expression in a dose-dependent manner (100 µM–200 µM, *p* < 0.001; Figure 2a). The expression of DHH and PTCH2 was not induced by low-dose (50 µM) palmitate in either 22Rv1 or ARCaP_M_ (*p* > 0.05; Supplemental Figure S1a,b). To examine specific transcriptomic changes due to cholesterol and free fatty acids, we used histone H3-Ac^K27^ chromatin immunoprecipitation (ChIP) sequencing. Examining known targets of Hh signaling associated with lineage plasticity, we found enrichment of the SOX2 promoter. Palmitate induced more H3-Ac^K27^ enrichment of SOX2 over either cholesterol or vehicle (Figure 2b). These findings were consistent with the concomitant induction of SOX2 expression by palmitate (100 µM), but not cholesterol in 22Rv1 (*p* < 0.001; Figure 2c). The induction of SOX2 by saturated fatty acids seems to involve autocrine Hh signaling.

Multiple pieces of evidence support the role of stromal fibroblastic cells in PCa progression and castration resistance [38,39,40,41,42]. Accordingly, we tested the impact of CAF on SOX2 expression in 22Rv1 cells in response to cholesterol and palmitate. The 100 µM palmitate concentration previously used seemed to induce CAF cell death; thus, SOX2 expression was tested under 50 µM palmitate, 20 µg/mL cholesterol, or a combination in cocultures of 22Rv1 and CAF. SOX2 expression in 22Rv1 under a lower palmitate dose was not observed (Supplemental Figure S1c). However, SOX2 expression in 22Rv1 was over twentyfold higher when cells were cocultured with CAF compared to the epithelia alone, under vehicle treatment (*p* < 0.01; Figure 2d). A further elevation of SOX2 expression was found with palmitate treatment compared to coculture control (*p* < 0.05; Figure 2d). Unexpectedly, the combination of palmitate and cholesterol dramatically promoted the 22Rv1 expression of SOX2, which was increased fourfold over cocultured CAF receiving no treatment (*p* < 0.01; Figure 2d). Conditional media collected from CAF treated with palmitate, in the presence or absence of cholesterol, was added to 22Rv1 cells. SOX2 was not induced in 22Rv1 under these conditions (Supplemental Figure S1d). This suggested that SOX2 expression by cancer epithelia was induced by crosstalk with CAF, rather than secreted factors from these cells. Both fatty acids and cholesterol seem to have independent roles in promoting Hh signaling. The role of Hh signaling in developmental processes suggests its potential efficiency in regulating differentiation through SOX2 regulation.

### 3.2. Combined Treatment of Palmitate and Cholesterol Induce Lineage Plasticity in PCa Cells by Cancer-Stroma Interactions

**Figure 3:**
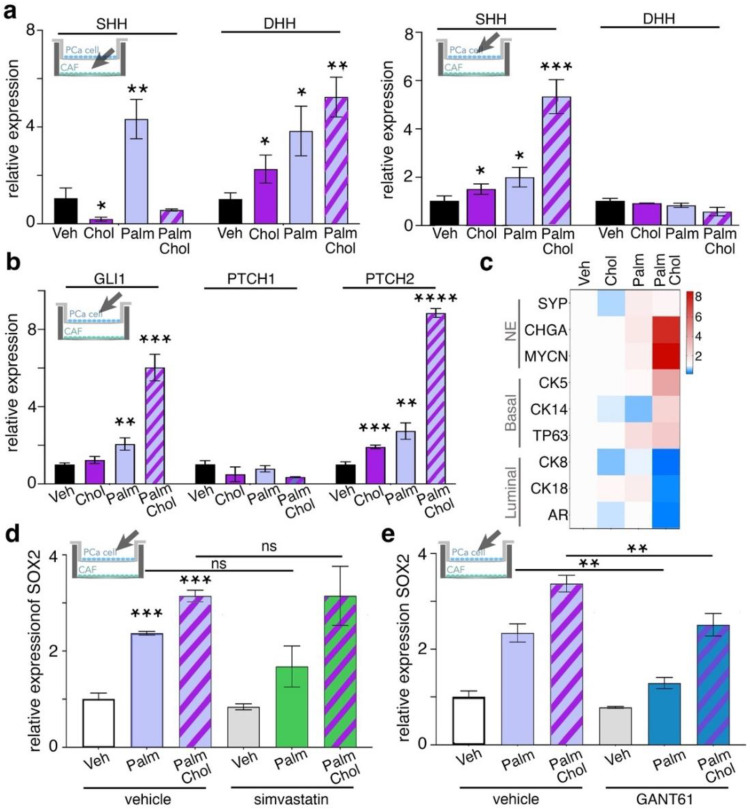
SOX2 expression in PCa cells is regulated by Hedgehog signaling. (**a**) SHH and DHH mRNA expression in CAF (cocultured with 22Rv1) or 22Rv1 (cocultured with CAF) was determined following treatment with palmitate, cholesterol, or both combined. (**b**) Gli1, PTCH1, and PTCH2 mRNA expression in 22Rv1 cocultured with CAF and treated with cholesterol, palmitate, or both in combination. (**c**) mRNA expression of lineage plasticity panel of genes in 22Rv1 treated with palmitate, cholesterol, or both in combination. (**d**) SOX2 mRNA expression in 22Rv1 cocultured with CAF and treated with palmitate or palmitate combined with cholesterol. These treatments were further supplemented with or without 5 nM simvastatin. (**e**) SOX2 mRNA expression in 22Rv1 cocultured with CAF treated with palmitate, palmitate combined with cholesterol, supplemented with or without 5 µM GANT61, Gli inhibitor. For all figure panels, mRNA expression in 22Rv1 cells was determined following treatment with 50 µM palmitate, combination of 50 µM palmitate and 20 µg/mL cholesterol, or vehicle for 48 h. Paired, 2-tailed *t* test: * *p* < 0.05, ** *p <* 0.01, *** *p* < 0.001, and **** *p* < 0.0001.

There are limited studies about the role palmitate and cholesterol play in stromal fibroblastic cells and how these metabolites affect PCa epithelia. Therefore, cocultures of PCa cells and primary CAF were incubated with palmitate in the presence or absence of cholesterol. The expression of Hh ligands in cocultured CAF and 22Rv1 was independently measured. We found that in CAF, both SHH and DHH were significantly elevated by the palmitate treatment alone, and the palmitate/cholesterol-combination treatment only further increased DHH (*p* < 0.01; Figure 3a). SHH expression was significantly elevated by the independent cholesterol and palmitate treatments when 22Rv1 and CAF were cocultured (*p* < 0.05; Figure 3a). SHH was further increased by the palmitate/cholesterol combination treatment (*p* < 0.001; Figure 3a). However, DHH was not found to be induced under these conditions in 22Rv1. When the two cell types were cocultured, the Hh signaling mediators GLI1 and PTCH2 were significantly increased by palmitate (*p* < 0.01) and palmitate/cholesterol-combination treatments (*p* < 0.001) as seen in 22Rv1 cells (Figure 3b). Since downstream SOX2 is reported to play key roles in PCa lineage plasticity, we next tested for the expression of luminal, basal, and neuroendocrine differentiation markers. The palmitate/cholesterol combination greatly elevated the expression of neuroendocrine and basal markers; but reduced the expression of luminal cell markers in 22Rv1 cells cocultured with CAF, an indicator of lineage plasticity (Figure 3c). Two-way ANOVA analysis of neuroendocrine-related genes, basal markers in 22Rv1 treated with palmitate/cholesterol combination, found them to be significantly increased compared to control cells (*p* < 10^−4^), while luminal markers were significantly increased compared to control (*p* < 10^−4^). In palmitate-treated cells, neuroendocrine markers were upregulated (*p* < 0.001) and luminal markers were downregulated (*p* < 0.01). The changes on the same genes were not evident by cholesterol alone. In testing the potential impact of low-density lipoprotein (LDL) cholesterol generated by palmitate metabolism, we treated cocultures with palmitate or a palmitate/cholesterol combination, and blocked HMG-CoA reductase with simvastatin. We found that these combinations did not decrease SOX2 expression in 22Rv1 cocultured with CAF (Figure 3d). Simvastatin also failed to reduce PTCH2 and GLI1 under palmitate treatment conditions (Supplemental Figure S1e). Thus, the upregulation of PTCH2 and DHH by palmitate was not cholesterol dependent, suggesting some specificity to Hh signaling by fatty acids. On the other hand, the administration of the Gli inhibitor, GANT61, was able to significantly limit SOX2 induction under either palmitate or palmitate/cholesterol combination treatments (*p* > 0.01, Figure 3e). The role of CAF in promoting PCa epithelial SOX2 expression in response to free fatty acid was dependent on Hh signaling rather than cholesterol biosynthesis.

**Figure 4:**
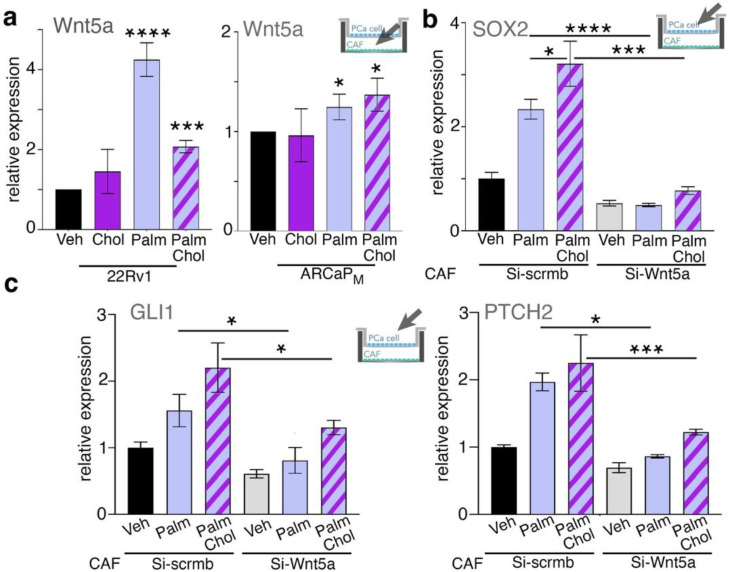
SOX2 expression in PCa cells is induced by CAF. (**a**) CAF-derived Wnt5a mRNA expression was determined following coculture with 22Rv1 or ARCaP_M_, having been treated with cholesterol, palmitate, and both in combination. (**b**) SOX2 mRNA expression in 22Rv1 cocultured with CAF following treatment with palmitate or palmitate and cholesterol in combination. CAF was subjected to transfection with either scrambled siRNA or Wnt5a siRNA. (**c**) Gli1 and PTCH2 mRNA expression in 22Rv1 was measured following coculture with CAF and treated with palmitate or palmitate and cholesterol. For all figure panels, mRNA expression in CAF and 22Rv1 cells were determined following treatment with 50 µM palmitate, combination of 50 µM palmitate and 20 µg/mL cholesterol, or vehicle for 48 h. Paired, 2-tailed *t* test: * *p* < 0.05, *** *p <* 0.001, and **** *p* < 0.0001.

In order to determine the mechanism of the elevated Hh-mediated SOX2 expression promoted by the CAF, compared to the PCa cells alone, we examined some putative mediators based on past reports suggesting the expression of Wnt ligands by CAF [40,42,52]. CAF cocultured with 22Rv1 treated with palmitate or a palmitate/cholesterol combination demonstrated significant Wnt5a expression by the CAF (*p* < 0.0001; Figure 4a). Similarly, the CAF demonstrated significant Wnt5a upregulation by palmitate alone and the palmitate/cholesterol combination over the untreated control when cocultured with ARCaP_M_ (*p* < 0.05; Figure 4a). Induction of Wnt2 and Wnt3a was not observed under the same conditions (Supplemental Figure S1f). To directly interrogate the role of Wnt5a, it was knocked down in CAF. Compared to the scrambled siRNA-control-transfected CAF, knocking down Wnt5a eliminated SOX2 induction in 22Rv1, otherwise stimulated by palmitate or the palmitate/cholesterol combination (*p* < 0.001; Figure 4b). In addition, knocking down Wnt5a expression in CAF significantly limited the 22Rv1 expression of Gli1 and PTCH2 under palmitate and palmitate/cholesterol-combination conditions (Figure 4c). The observed cooperativity of prostatic epithelia and CAF in response to palmitate was lost when Wnt5a signaling was limited.

### 3.3. Free Fatty Acid Signaling Mediates PCa Androgen Targeted Therapy Sensitivity

**Figure 5:**
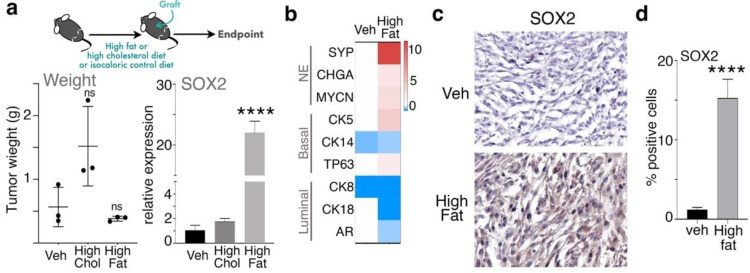
SOX2 expression is promoted under high-fat diet in tumors xenografted in mice. (**a**) NSG mice were randomly divided into 3 groups, pretreated with isocaloric, high-fat (40%), high-cholesterol (2%), or isocaloric rodent diet as vehicle control (Veh) for one month (n = 3). Cell recombinants were prepared by mixing 2.5 × 10^5^ epithelial (ARCaP_M_) cells with 7.5 × 10^5^ CAF in collagen. Orthotopic grafting was constituted by placing the collagen plugs in the 2 anterior lobes of the prostate. Mice were sacrificed 1 month later, and tumors were excised. Tumor weight of each group was measured (ns = not significant). SOX2 mRNA expression in tissues from mice treated with vehicle, cholesterol, or high-fat diet was detected by qPCR. (**b**) Lineage plasticity gene-panel expression was tested in tissues in mice from the indicated treatment conditions. (**c**) SOX2 expression in tumor tissues was determined by immunohistochemistry (magnification × 200). (**d**) The bar graph shows quantification of the percentage of SOX2-positive cells per field by immunohistochemical staining based on at least 5 fields from each of the 3 specimens per treatment group. Paired, 2-tailed *t* test: **** *p <* 0.0001.

In light of previous studies examining the role of cholesterol and fatty acids on PCa tumor growth, we tested the role of high-fat versus high-cholesterol isocaloric diets in prostatic orthotopic mouse models engrafted with ARCaP_M_ and CAF cells. The mouse weight did not differ among the control, high-fat, and high-cholesterol conditions (n = 3, Supplemental Figure S2a). There was no statistical difference in tumor weight among the groups, although the tumor weight was somewhat greater in the mice given a high cholesterol diet, compared to the control or high-fat diet (Figure 5a). These findings were corroborated using colony-forming assays of both 22Rv1 and ARCaP_M_ cells grown with palmitate, cholesterol, or a combination thereof. Cholesterol promoted greater colony formation compared to the control in both cell types (*p* < 0.0001; Supplemental Figure S2b,c). Strikingly, the RNA expression of SOX2 was about twentyfold greater in the tumors of the high-fat-diet-fed mice compared to cholesterol or control (*p* < 0.0001; Figure 5a). Further induction of the lineage plasticity markers of neuroendocrine and basal differentiation was greatest under high-fat diet condition (*p* < 10^−4^, *p* < 0.01, respectively), while luminal markers were significantly decreased (*p* < 10^−4^, Figure 5b). SOX2 histochemical staining was much greater in tumor tissues from mice on the high-fat diet than those on the control isocaloric diet (Figure 5c). While changes in mouse diet did not appreciably effect tumor growth, the high-fat diet potentiated SOX2 expression and the downstream lineage plasticity of the tumors.

**Figure 6:**
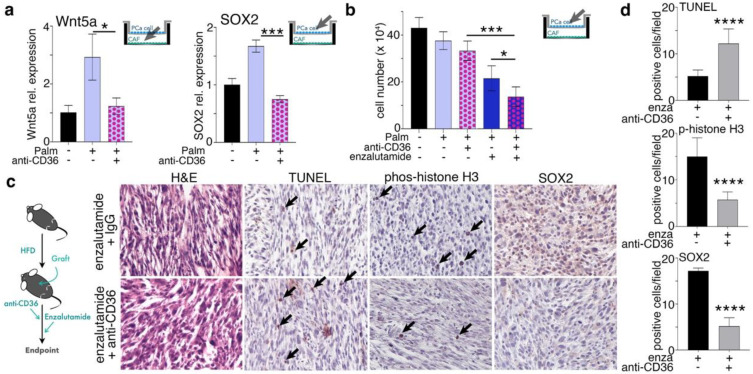
SOX2 expression is inhibited by anti−CD36 antibody under high-fat diet. (**a**) Wnt5a mRNA expression in CAF cocultured with 22Rv1 and treated with palmitate or palmitate and CD36 neutralizing antibody. SOX2 mRNA expression in 22Rv1 cocultured with CAF and treated with palmitate or palmitate and CD36 neutralizing antibody. (**b**) Cell counts of 22Rv1 cocultured with CAF and treated with palmitate, CD36 neutralizing antibody, enzalutamide and combinations of each. (**c**) Hematoxylin and eosin (H&E) staining of tumor tissues was followed by IHC for SOX2, TUNEL, or phosphorylated histone H3 (magnification × 200).(**d**) The bar graph shows quantification of the number of phosphorylated histone H3− and TUNEL−positive nuclei per field, and the percentage of SOX2-positive cells per field by immunohistochemical staining, n > 5. For all figure panels, mRNA expression in CAF and 22Rv1 cells was determined following treatment with 50 µM palmitate, combination of 50 µM palmitate and 20 µg/mL cholesterol, or vehicle for 48 h. N = 4. Paired, 2-tailed *t* test: * *p* < 0.05, *** *p <* 0.001, and **** *p <* 0.0001.

We next used anti-CD36 neutralizing antibody to inhibit fatty acid signaling under palmitate treatment. Mice were treated with anti-CD36 antibody for one week, since neutralizing antibodies generally take one week to achieve a dose that reaches biologic efficacy. It is reported that maximal apoptosis following castration in mice occurred on day four [53,54,55,56]. Thus, we treated mice with anti-CD36 for one week to limit free fatty acid signaling, then administered enzalutamide for three days and harvested the next day. We found that anti-CD36 antibody treatment limited the expression of Wnt5a by CAF and SOX2 expression by PCa epithelia (*p* < 0.001; Figure 6a). In addition, a cell-counting study revealed that inhibiting CD36 alone had little effect on cell proliferation, while the combination of anti-CD36 and enzalutamide resulted in fewer PCa cells compared to enzalutamide alone (*p* < 0.05; Figure 6b). We tested the role of anti-CD36 in mice subcutaneously implanted with 22Rv1 and CAF under high-fat diet conditions. A mouse study was designed based on the findings of the coculture experiments where all mice were given a high-fat diet and xenografted with 22Rv1 and CAF cells. When the tumors reached approximately 1 cm^3^, the mice were randomized to receive anti-CD36 neutralizing antibody or IgG followed by enzalutamide (Figure 6c). Consistent with the coculture studies, SOX2 expression was inhibited. We also found phosphorylated-histone H3 was inhibited, while TUNEL staining was elevated by anti-CD36 antibody in the context of enzalutamide, compared to the IgG control.

## 4. Discussion

We identified the role of saturated fatty acids in PCa-cell lineage plasticity reprogramming via paracrine interactions with CAF. Initially, our data confirmed that exogenously added cholesterol could activate canonical Hh signaling associated with the cilium (Figure 1). Interestingly, we found the ARCaP_M_ cells to be more sensitive to palmitate compared to the 22Rv1 cell line. We initiated this study with 22Rv1 and ARCaP_M_, because these two cell lines maintain some level of sensitivity to androgen-targeted therapy and can be demonstrated to be more resistant or sensitive to androgen-targeted therapy. Palmitate induced a Gli-independent Hh signaling pathway (Figure 2). This finding represented an observed effect of non-canonical signaling in PCa cells. When PCa cells were cocultured with CAF, we found Hh signaling was induced more by palmitate, in the presence or absence of cholesterol, compared to the cholesterol-treated group (Figure 3). Palmitate-induced Hh signaling was found to promote SOX2 in PCa epithelia in the context of CAF as a master regulator of lineage plasticity [16]. SOX2 is also known to promote cell transition from AR-dependent luminal epithelial cells to AR-independent basal-like cells [46]. Previous reports have demonstrated that reduced expression of the luminal phenotype correlates with poor survival prognosis in PCa patients [42,57,58].

Our findings support these studies, as the enhanced SOX2 expression seen during palmitate and cholesterol treatments also contributed to increased basal and neuroendocrine gene expression. Palmitate, alone or in combination with cholesterol, elevated the expression of basal and neuroendocrine markers, while downregulating the expression of luminal cell markers. The translational implications of PCa lineage plasticity are well described as a means of therapy-resistance development. However, the upstream mediators for this differentiative state are less understood. Although Hh signaling is a viable upstream mediator of SOX2 expression, the observation that CAF can further promote SOX2 expression and lineage plasticity was an important finding in revealing a new interventional target mitigating the response to a high-fat diet.

The combination of palmitate and cholesterol induced the highest levels of SOX2 in the context of CAF. This indicated that CAF plays an important role in the expression of SOX2 in epithelia cancer cells. Our previous research found that prostate stroma could induce PCa-cell proliferation and tumorigenesis by CAF-derived Wnt ligands [40]. Many studies have demonstrated that the non-canonical Wnt pathway plays an important role in PCa metastasis [59,60,61]. Androgen-signaling inhibition elevated Wnt2, Wnt3a, and Wnt5a expression in CAF, and enhanced tumor epithelial cell survival [62]. Other studies indicated that Wnt5a expression in the circulating tumor cells of patients with metastatic castration-resistant PCa was a negative indicator of overall survival [63]. In addition, Wnt5a expression by bone-marrow fibroblasts is found to promote PCa bone metastasis [64]. Antagonizing Wnt5a in CAF can inhibit gastric-cancer-cell growth and migration [65]. Our studies here support Wnt5a as a potential paracrine driver of tumor progression in response to a high-fat diet (Figure 4). We found that Wnt5a from CAF can drive Hh-mediated SOX2 expression in PCa cells to promote lineage plasticity. Knowing the prevalence of PCa and the consumption of high-fat diets in the western world, our present findings warrant further clinical validation.

A commercially available Gli inhibitor, GANT61, was able to inhibit SOX2 induction in PCa epithelia when cocultured with CAF treated with palmitate. However, in the same context, there was no difference in the expression of SOX2 when simvastatin was given instead. This indicates that the role of CAF in promoting PCa epithelial SOX2 expression in response to free fatty acid is associated with Hedgehog signaling rather than cholesterol biosynthesis. A recent study showed that SOX2 expression correlated with a shorter time to metastasis and decreased survival after biochemical recurrence in a case-control cohort study of 1028 annotated tumor specimens [66]. They also demonstrated that SOX2 mediates metabolic reprogramming of prostate cancer cells by inducing increased glycolysis and glycolytic capacity, as well as increased numbers of mitochondria [66].

**Figure 7:**
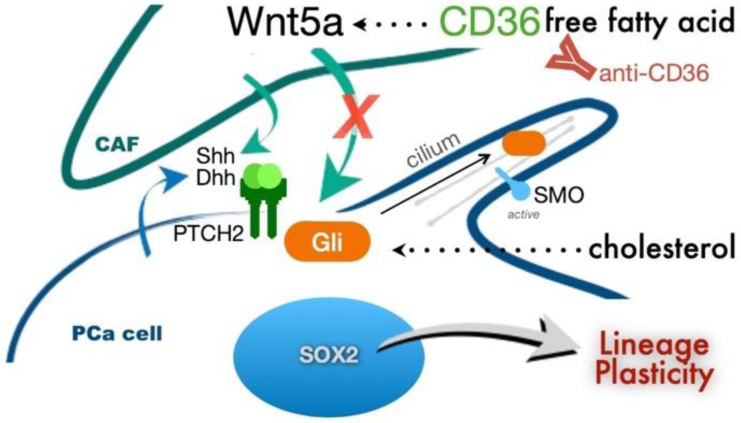
SOX2 expression and lineage plasticity of PCa epithelia supports resistance to androgen-targeted therapy. SOX2 expression in PCa cells is regulated by Hedgehog signaling. Knockdown Wnt5a in CAF could inhibit Hedgehog signaling and SOX2 expression in PCa epithelia. The inhibition of palmitate signaling by anti-CD36 antibody limits the Wnt5a expression in CAF, inhibiting SOX2 expression in PCa treated with both palmitate and enzaltamide.

CD36 plays an important role in the process of metastasis and correlates with poor prognosis in oral cancer, lung squamous cancer, bladder cancer, and breast cancer [67]. Inhibiting CD36 in PCa could reduce the uptake of fatty acids, cell proliferation, and cancer aggressiveness [68]. Our data suggest that CD36 inhibition could further reduce Wnt5a expression by CAF, inhibit SOX2 expression in PCa, inhibit proliferation, and induce apoptosis in mice receiving a high-fat diet and treated with enzalutamide (Figure 6 and Figure 7). Thus, targeting CD36 might be an effective strategy for treating PCa patients.

Obesity in PCa patients, associated with excessive white adipose tissue, contributes to the population of CAF and promotes cancer progression [69]. Interestingly, our studies involving NSG (NOD SCID gamma) mouse xenograft models have a limited capacity to become obese. We are aware that the results of a high-fat diet on the mouse models may need to be further studied in immunocompetent models. Insulin insensitivity associated with obesity downstream of PI3K-mTOR signaling promotes lipogenesis [70,71,72]. It may play a role in remodeling the tumor microenvironment as well as support anti-apoptotic signals in the cancer cells. NSG mice are known to exhibit insulin-dependent diabetes and the high-fat diet prevents the mice from developing autoimmune diabetes [73]. As such, the standard elevation of insulin associated with a high-fat diet is not observed in NSG mice. Thus, the observations in our work are likely due to a more direct result of circulating fat (and cholesterol), akin to that observed in cell culture. Under the high-fat diet, the elevated circulating fatty acids likely supported CD36 signaling of paracrine initiators such as Wnt5a by stromal fibroblasts. While further exploration will be necessary, our data indicate that palmitate promotes paracrine Wnt5a secretion from CAF to induce PCa lineage plasticity and therapy resistance.

## 5. Conclusions

The present study indicates that palmitate and cholesterol induce lineage plasticity in prostate cancer by cancer–stroma interactions through Hedgehog and non-canonical Wnt signaling. We found that a high-fat diet induces lineage plasticity in prostate cancer epithelial cells by increasing SOX2 expression in both coculture and xenograft models. The resulting palmitate signaling promotes lineage plasticity culminating in resistance to androgen-targeted therapy. Understanding paracrine and autocrine interactions of Hedgehog and androgen signaling will enable the restoration of treatment responsiveness.

## Data Availability

All data presented in the study are available from the corresponding author by request.

## Supplementary Materials

The following supporting information can be downloaded at: https://www.mdpi.com/article/10.3390/cancers14143449/s1, Figure S1. (a,b) The expression of Dhh and PTCH2 in 22Rv1 and ARCaP_M_ were measured by rtPCR following palmitate treatment. (c) SOX2 mRNA expression in 22Rv1 were treated with palmitate (50 µM), cholesterol (20 µg/mL), palmitate and cholesterol combination treatment, or NT. (d) SOX2 mRNA expression in 22Rv1 treated with CAF-conditioned media alone (NT), supplemented with palmitate, cholesterol, or palmitate and cholesterol combination treatment. (e) Gli1 and PTCH2 mRNA expression in 22Rv1 cocultured with CAF and treated as indicated inclusive of simvastatin. (f) Wnt2 and Wnt3a mRNA expression by CAF cocultured with 22Rv1 following indicated treatments. Cells were treated for 48 h in complete media, unless noted. Figure S2. (a) NSG mice were randomly divided into three groups, pretreated with an isocaloric high-fat (40%) diet, high-cholesterol (2%) diet, and rodent diet for one month. Cell recombinants were prepared by mixing 2.5 × 10^5^ epithelial (ARCaP_M_) cells with 7.5 × 10^5^ cancer-associated fibroblasts in collagen. Orthotopic grafting constituted the placing of the collagen plugs in the two anterior lobes of the prostate. Mice were sacrificed 1 month later, and tumors were excised. Bar graphs show mouse weight (a). (b,c) 22Rv1 and ARCaP_M_ were plated in a clonogenic survival assay, treated with 50 µM palmitate, 20 µg/mL cholesterol, and the combination of both. Colonies were stained with crystal violet 2 weeks later. Quantification of colonies were shown by optical density 595 (OD_595_) measured by spectrophotometer in three independent experiments. Paired, two-tailed *t* test: **** *p* < 0.0001. Table S1. Real-time PCR Oligonucleotide Sequence. Table S2. Formulation of high fat diet and high cholesterol diet.
