# Supplementary material for: Fatty Acid Signaling Impacts Prostate Cancer Lineage Plasticity in an Autocrine and Paracrine Manner
Source: Cancers (Basel). 2022 Jul 15;14(14):3449. doi: 10.3390/cancers14143449 (PMC9318639; doi:10.3390/cancers14143449)
Supplement: Supplementary file 1 [file cancers-14-03449-s001.zip › Supplement Figures.pdf]

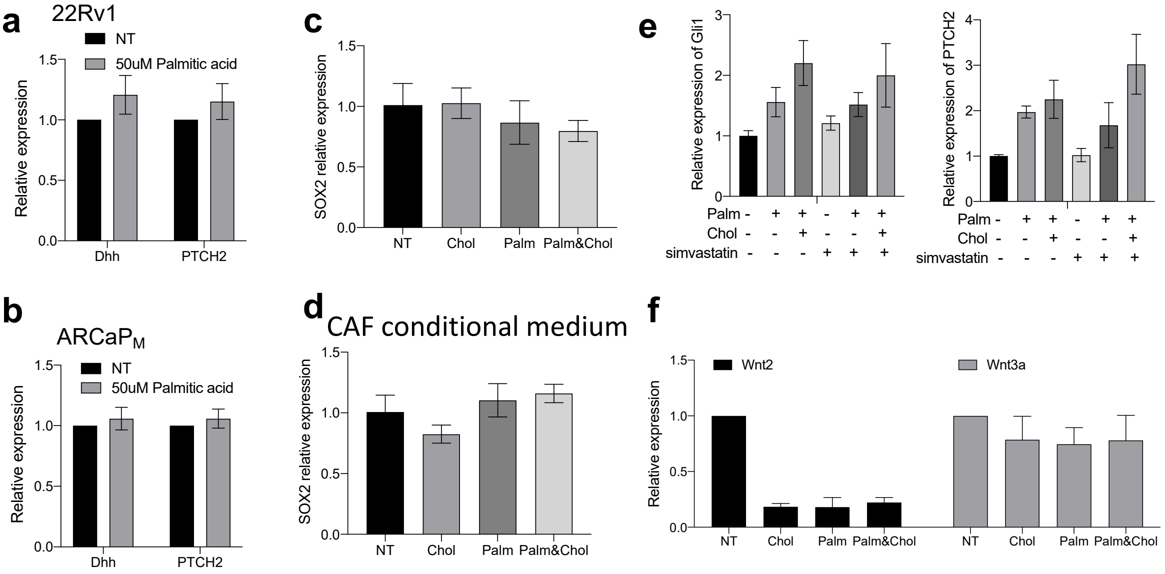

Figure S1. a,b) The expression of Dhh and PTCH2 in 22Rv1 and ARCaP$_M$ were measured by rtPCR following palmitate treatment. c) SOX2 mRNA expression in 22Rv1 were treated with palmitate (50 μM), cholesterol (20 μg/mL), palmitate and cholesterol combination treatment, or NT. d) SOX2 mRNA expression in 22Rv1 treated with CAF conditioned media alone (NT), supplemented with palmitate, cholesterol, or palmitate and cholesterol combination treatment. e) Gli1 and PTCH2 mRNA expression in 22Rv1 co-cultured with CAF and treated as indicated inclusive of simvastatin. f) Wnt2 and Wnt3a mRNA expression by CAF co-cultured with 22Rv1 following indicated treatments. Cells were treated for 48 hours in complete media, unless noted.

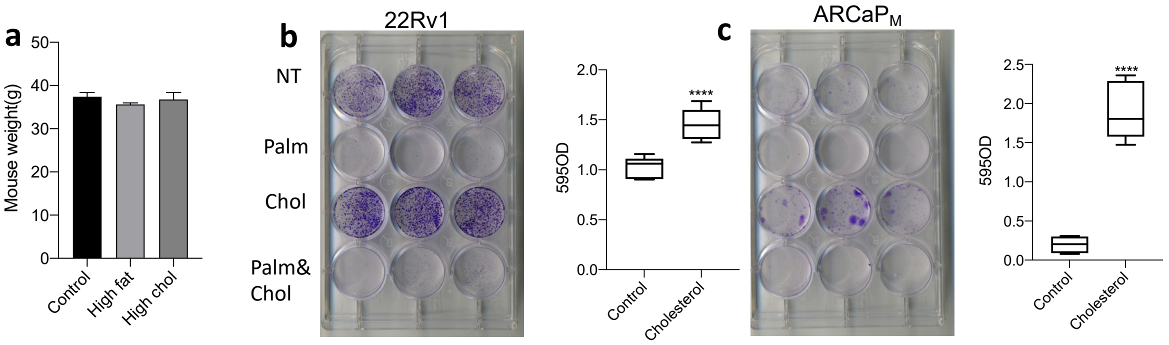

Figure S2. a) NSG mice were randomly divided into 3 groups, pretreated with isocaloric high fat (40%) diet, high cholesterol (2%) diet, and rodent diet for one month. Cell recombinants were prepared by mixing 2.5×10^5 epithelial (ARCaP$_M$) cells with 7.5×10^5 cancer-associated fibroblasts in collagen. Orthotopic grafting constituted the placing of the collagen plugs in the 2 anterior lobes of the prostate. Mice were sacrificed 1 month later, and tumors were excised. Bar graphs show mouse weight a).
b, c) 22Rv1 and ARCaP$_M$ were plated in a clonogenic survival assay, treated with 50 μM palmitate, 20 μg/mL cholesterol, and the combination of both. Colonies were stained with crystal violet 2 weeks later. Quantification of colonies were shown by optical density 595 (OD$_{595}$) measured by spectrophotometer in three independent experiments. Paired, 2-tailed t test: **** $p < 0.0001$.
