# Supplementary material for: Fatty Acid Signaling Impacts Prostate Cancer Lineage Plasticity in an Autocrine and Paracrine Manner
Source: Cancers (Basel). 2022 Jul 15;14(14):3449. doi: 10.3390/cancers14143449 (PMC9318639; doi:10.3390/cancers14143449)
Supplement: Supplementary file 1 [file cancers-14-03449-s001.zip › Supplement Table1.pdf]

Table S1. Real-time PCR Oligonucleotide Sequence.

Gene	Forward Primer Sequence	Reverse Primer Sequence
**Gli1**	GGGTGCCGGAAGTCATACTC	GCTAGGATCTGTATAGCGTTTGG
**Gli2**	CTGCCTCCGAGAAGCAAGAAG	GCATGGAATGGTGGCAAGAG
**Gli3**	TGGTTACATGGAGCCCCACTA	GAATCGGAGATGGATCGTAATGG
**PTCH1**	CCAGAAAGTATATGCACTGGCA	GTGCTCGTACATTTGCTTGGG
**PTCH2**	GCTTCGTGCTTACTTCCAGGG	CATGCGGAGACCTAATGCCA
**SHH**	GGACAGGCTGATGACTCAGA	GCCCTCGTAGTGCAGAGACT
**IHH**	GCTCACCCCCAATTACAATC	AGATAGCCAGCGAGTTCAGG
**DHH**	TGATGACCGAGCGTTGTAAG	GCCAGCAACCCATACTTGTT
**SYP**	TCGGCTTTGTGAAGGTGCTGCA	TCACTCTCGGTCTTGTTGGCAC
**CHGA**	TAAAGGGGATACCGAGGTGATG	TCGGAGTGTCTCAAAACATTCC
**MYCN**	TGATCCTCAAACGATGCCTTC	GGACGCCTCGCTCTTTATCT
**CK5**	AGGAGTTGGACCAGTCAACAT	TGGAGTAGTAGCTTCCACTGC
**CK14**	TGAGCCGCATTCTGAACGAG	GATGACTGCGATCCAGAGGA
**TP63**	CCACCTGGACGTATTCCACTG	TCGAATCAAATGACTAGGAGGGG
**CK8**	CAGAAGTCCTACAAGGTGTCCA	CTCTGGTTGACCGTAACTGCG
**CK18**	TCGCAAATACTGTGGACAATGC	GCAGTCGTGTGATATTGGTGT
**AR**	CCAGGGACCATGTTTTGCC	CGAAGACGACAAGATGGACAA
**Wnt5a**	ATTCTTGGTGGTCGCTAGGTA	CGCCTTCTCCGATGTACTGC
**SOX2**	GAGAGAAAGAGGAGAGAGAAA	GCCGCCGATGATTGTTATTATT
**TBP**	CCACTCACAGACTCTCACAAC	CTGCGGTACAATCCCAGAACT
