# Supplementary material for: Fatty Acid Signaling Impacts Prostate Cancer Lineage Plasticity in an Autocrine and Paracrine Manner
Source: Cancers (Basel). 2022 Jul 15;14(14):3449. doi: 10.3390/cancers14143449 (PMC9318639; doi:10.3390/cancers14143449)
Supplement: Supplementary file 1 [file cancers-14-03449-s001.zip › Supplement Table2.pdf]

Table S2. Formulation of high fat diet and high cholesterol diet.

40% kcal Fat, 3.41kcal/gram	
**Ingredient**	**gram/kg**
Maltodextrin	143.7
Sucrose	160
Lard	152
Casein	228
Cellulose	263
DL Methionine	2
Choline Chloride	1.3
Vitamin Mix	10
Mineral Mix	40
Cholesterol	0
Total	1000

Picolab 5053, 2% cholesterol	
**Ingredient**	**gram/kg**
Picolab 5053	980

40% kcal Fat, 3.41kcal/gram			
	gram%	kcal/gram	%kcal
Protein	20.5	0.82	24.0
Fat	15.2	1.37	40.1
Carbohydrate	30.6	1.23	35.9
Cellulose	26.3	3.41	100
Cholesterol	0	0	0

Picolab 5053, 2% cholesterol			
	gram%	kcal/gram	%kcal
Protein	20.0	0.80	23.4
Fat	5.6	0.50	14.8
Carbohydrate	52.9	2.11	61.8
Cellulose	4.7	3.41	100
Cholesterol	2		
